# Search for Wives

**Published:** 1877-03

**Authors:** 


### SEARCH FOR WIVES.

Where do men usually discover the women who afterwards become their wives?—is a question we have occasionally heard discussed, and the custom has invariably become of value to young lady readers. Chance has much to do in the affair, but then there are important and governing circumstances. It is certain that few men make a selection from ball-rooms, or any other places of public gayety, and nearly as few are influenced by what may be called "showing off" in the streets, or by any allurements of dress. Our conviction is, that ninety-nine hundred parts of all the finery with which women decorate or load their persons go for nothing, as far as husband-catching is concerned. Where, and how, then, do men find their wives? In the quiet homes of their parents or guardians, at the fireside, where the domestic graces and feelings are alone demonstrated. These are the charms which most surely attract the high as well as the humble. Against these, all the finery and airs in the world sink into insignificance.

It is with pleasure that we announce to our readers that Dr. Rae has resumed practice, and opened offices in the Bible House, rooms 51 and 51½, entrance on 3d Avenue. Some of the cures of chronic complaints effected by Dr. Rae while in practice were astonishing and almost incredulous, especially in cases of cancer, scrofulosis, paralysis, and female complaints.

